# On-line training and testing of knowledge of trial protocols to facilitate centre start-up and reduce protocol deviations

**DOI:** 10.1186/1745-6215-14-S1-P57

**Published:** 2013-11-29

**Authors:** Martin Dennis, Rustam Al Shahi Salman, Gillian Mead, David Perry

**Affiliations:** 1 University of Edinburgh, Edinburgh, UK

## 

In multicentre trials, the chief investigator and sponsor, have a responsibility to ensure that researchers in each centre have sufficient knowledge of the trial protocol to ensure patients' safety and minmise protocol deviations. However, this can involve literally hundreds of individuals spread across a wide geographical area. Delivering face-to-face training, and evaluations of knowledge, is impractical and unaffordable, especially for academically led non-commercial trials. In the completed CLOTS trials (http://www.clotstrial.com) (recruited 2876 pts in 107 UK centres 2001 to 2012) we provided on-line access, via our trial website, to streamed lectures on the trial protocol and procedures and tested researchers knowledge with an on-line quiz. Successful completion of the quiz enabled the researcher to print off a personalised certificate.

In the recently started FOCUS (http://www.focustrial.org.uk) and RESTART (http://www.RESTARTtrial.org) trials we have further developed this concept. We provide access via our website to a series of narrated Powerpoint presentations, which can be either viewed through the website or downloaded. Each Powerpoint presentation has an associated on-line quiz. Each quiz contains 10 questions, of which one can only be answered if the person has actually viewed the Powerpoint i.e. it relates to a specific piece of information embedded in the slides. This was inserted to increase the likelihood of the researcher actually completing the presentations. The researcher's IT account cannot be activated until the quiz has been completed.

This development helps to ensure adequate knowledge of trial procedures and facilitates rapid trial expansion with remote site initiation visits.

